# *Leporinus elongatus* induced spawning using carp pituitary extract or mammalian GnRH analogue combined with dopamine receptor antagonists

**DOI:** 10.21451/1984-3143-2017-AR983

**Published:** 2018-08-16

**Authors:** Thiago Scremin Boscolo Pereira, Camila Nomura Pereira Boscolo, Renata Guimarães Moreira, Sergio Ricardo Batlouni

**Affiliations:** 1 , ,; 2 Medical School FACERES, , ,; 3 University of Sao Paulo (USP), , , .; 4 Sao Paulo State University (UNESP), , , .

**Keywords:** final maturation, gonadal steroids, hormonal treatment, ovulation, spawning performance

## Abstract

Several studies have been developed to support the replacement of the crude carp pituitary extract (CPE) by synthetic products for induced reproduction of South American rheophilic species. However, results have been quite heterogeneous and there is no consensus or a routine use of synthetic products in these species. Thus, the aim of this study was to evaluate the ovulatory process in *L. elongatus* using different protocols of hormonal induction. Thus, fifteen wild mature females maintained at the Experimental Fish Station, Salto Grande, SP, Brazil were submitted to three different hormonal treatments: CPE (fractioned dose: 0.5 and 5.0 mg kg^-1^); mGnRHa (single dose: 3.5 µg kg^-1^) and mGnRHa (single dose: 5.0 µg kg^-1^). The spawning rate and absolute fecundity were similar among the treatments, but fertility rates were higher for CPE treatment (23.60 ± 9.40) then for mGnRHa treatments (close to or zero zero). Although females ovulated in all treatments, none of them provided viable embryos, showing hatching rates close to zero or zero. Both mGnRHa treatments were more potent for inducing the ovulatory process then CPE treatment, which was evidenced by the fact that the formers showed higher volume density of postovulatory follicles (POF). Accordingly, E_2_ and 17α-OHP plasma levels were higher for the mGnRHa treated females compared to the CPE one at the time of ovulation. In this study we confirmed previous scientific evidence that, regardless of whether promoting ovulation, the use of conventional CPE and GnRH doses are not appropriate for some South American migratory species, due to the non-attainment of viable embryos. Moreover, we have brought new information about the relationship between reproductive performance and gonadal steroids concentrations using different hormonal therapies, contributing to understand the reasons for *Leporinus elongatus* embryo loss in induced spawning.

## Introduction

*Leporinus elongatus* is a medium-sized, total spawner rheophilic fish, commercially relevant and known to be a good model for studies concerning the reproductive biology of rheophilic species (Duke Energy International-Geração Paranapanema S/A., 2003). Moreover, this species was one of the ten most produced fish in Brazil in 2014 ( IBGE, 2014 ), mainly due to its high quality meat (Duke Energy International-Geração Paranapanema S/A., 2003) and acceptance for commercial, subsistence, and sport fishing purposes ( Petrere *et al*., 2002 ; Giamas and Vermulm-Jr, 2004 ). It is similar to other tropical rheophilic fish which perform a reproductive migration in order to have a total spawning in their native habitat. However, when kept in captivity, even though they reach advanced stages of gonadal development, the final maturation and ovulation does not occur ( Godinho, 2007 ; Makrakis *et al*., 2007 ; Brito and Carvalho, 2013). Therefore, this rheophilic species needs to be hormonally induced for providing fingerlings under captivity conditions ( Sato *et al*., 2000 ; Streit-Jr *et al*., 2008 ).

In this concern, the use of Gonadotrophin releasing hormones analogues (GnRHa) has grown rapidly due to its numerous advantages, but mainly because they are not species-specific molecules and present high structural similarities among fish. Moreover, due to their synthetic nature, they pose no risk of transmitting diseases, such as carp pituitary extracts (CPE) do, and since they act at higher levels of the hypothalamic-pituitary gonad axis, they stimulate the release of LH and FSH, as well as other pituitary hormones, which may serve important reproductive functions (review in Mylonas *et al*., 2010 ). However, although the efficiencies of the use of synthetic products in some South American rheophilic species (Ittze’s *et al*., 2015; Viveiros *et al*., 2015 ) have already been shown - such as obtaining ovulation and viable embryos - and even if they sometimes have a higher potential for inducing ovulation compared to that of CPE ( Pereira *et al*., 2017 ), the use of mGnRH in these species is frequently associated with ovulation failure ( Carneiro and Mikos, 2008 ) and / or failure in obtaining viable embryos ( Acuña and Rangel 2009 ; Paulino *et al*., 2011 ; Pereira *et al*., 2017 ).

Therefore, the most commonly used technique for obtaining viable embryos from South American rheophilic species is still the hypophysation with the application of CPE (0.5 and 5.0 mg kg^ -1^) ( Caneppele *et al*., 2015 ; De Souza *et al*., 2015 ; Ittzés *et al*., 2015 ; Viveiros *et al*., 2015 ; Schorer *et al*., 2016 ; Pereira *et al*., 2017 ). Nevertheless, the main problem related to the use of CPE is a constant uncertainty and unpredictability of a successful ovulation ( Criscuolo–Urbinati *et al*., 2012 ; Hainfellner *et al*., 2012a ; 2012b ; Pereira *et al*., 2017 ). In the specific case of *L. elongatus*, the low potential for inducing ovulation, the heterogeneous results concerning fertility rates and the number of oocytes that are retained in the post-spawning ovaries using CPE are highlighted by Sato and collaborators (2000). A high proportion of oocytes retained in the ovaries after stripping (week ovulation) also seems to be a constant in treatments using CPE in South American rheophilic species ( Sato *et al*., 2000 ; Hainfellner *et al*., 2012a ; 2012b ; Criscuolo–Urbinati *et al*., 2012 ; Pereira *et al*., 2017 ). Thus, in this study we aimed at improving *L. elongatus* reproductive performance by using low doses of mGnRHa and then comparing the evolution of the final maturation and ovulation obtained through this treatment with that obtained through the use of conventional CPE doses.

## Materials and methods

### Broodstock maintenance

Wild males and females (at a sex ratio of 1:1) were maintained at Duke Energy’s Experimental Fish Station (22 ° 54'23 .81 “S and 50 ° 00 '05.06” W). The animals were kept in 200 m^2^ ponds (ca. 0.25 fish m^2^) under average temperature of 27°C and natural photoperiod. Fish were manually fed a pelleted balanced commercial diet (moisture content 10.0%; crude protein 32.0%; ethereal extract 10.0%; fibrous matter 7.0%; ash 10.0%; calcium 1.2%; phosphorus 1.2%) corresponding to 3.0% of total body weight twice a day.

### Experimental Protocols

Fifteen mature females with a mean biomass of (mean ± SEM) 1.5 + 0.4 kg were randomly selected for the induced breeding experiments. At the time of spawning, broodstock were transported to the lab for acclimatization and maintained in 500 L tanks with constant water circulation. Females were randomly submitted to three different hormonal treatments: 1-) crude carp pituitary extract (CPE) (Fish braz®) – two doses (0.5 and 5.0 mg kg^-1^, 12h interval, diluted in 0.5 mL saline - 0.9%); 2-) mammalian gonadotropin-releasing hormone analogue (mGnRHa) (conceptal ® / Intervet) – single dose (3.5 µg kg ^-1^, diluted in 10 ml sterile buffered diluent, associated with a dopamine inhibitor (10 mg kg^-1^ metoclopramide, diluted in 0.5 ml saline - 0.9%) and 3-) mammalian gonadotropin-releasing hormone analogue (mGnRHa) (conceptal ® / Intervet) – single dose (5.0 µg kg^-1^, diluted in 10 ml sterile buffered diluent, associated with a dopamine inhibitor (10 mg kg^-1^ metoclopramide, diluted in 0.5 ml saline - 0.9%).

### Reproductive performance

The latency period was defined as the time between the first injection and fish ovulation for CPE treatment percentage of spawning females was determined using the following formula: total number of spawned females/total number of induced females x 100. The total number of oocytes released by each female (absolute fecundity) was estimated according to the method proposed by Pereira *et al*. (2017) . After that, oocytes of each female were fertilized using a pool of semen from males from the same broodstock. To avoid the effects of factors unrelated to the influence of the females during the artificial breeding process, the same pool of semen was used for all treatments. Approximately 0.5 mL semen was used to fertilize 50 g of oocytes.

Soon after fertilization, ~50 ml of eggs from each female were distributed into four funnel-shaped plastic incubators with a capacity of 18 L. Then, 18 g of eggs were placed in each incubator with a constant water flow of 5 L/min^-1^. To determine the fertilization rate, 8-12 hours post-fertilization, 100 eggs from each female were randomly sampled and counted, and those which were normally dividing were scored (viable embryos). After 17 post-fertilization, overall hatching rate was determined by counting the number of hatched eggs/number of fertilized eggs X 100.

All spawned females were euthanized after spawning with an overdose of anesthesia (2 g ethylaminobenzoate: 150 mL alcohol: 20 L water) and had their ovaries collected. The gonadosomatic index (GSI) was determined according to Criscuolo-Urbinati *et al.* (2012) and Pereira *et al.* (2017) proposed formulae for calculating this index in post-spawning females.

Water parameters were measured throughout the latency period and egg incubation (measured every experimental day at 09:00 h) using a YSI model 55 oximeter and a YSI model 63 multiparameter sonde (Yellow Spring Instruments, Yellow Springs, OH, USA). The experiments were performed under natural photoperiod and average water mean temperature, pH and dissolved oxygen were respectively 26.5 ± 1.25°C, 6.9 ± 0.4 and 6.03 ± 0.54 mg L^ -1^.

### Histomorphometric analyses

For the histological evaluation (volume density), cranial, medial, and tail regions of the ovary tissues were fixed in Bouin solution for routine histological procedures, according to the metodology applied by Pereira *et al.* (2017)

The volume density was determined using light microscopy and a 320-intersection grid. Three fields from each region of the ovary (anterior, medial, and cranial; total of nine fields) were randomly selected, with a total of 2880 points scored for each animal at magnification X 4. For this analysis, the methodology applied by Pereira *et al*. (2017) , was used with some modifications. Points were classified as one of the following: previtellogenic oocyte (PV), cortical alveoli oocyte (CA), immature oocytes with incomplete vitellogenesis and cytoplasm not fully filled by yolk (IV), mature vitellogenic oocytes with cytoplasm filled entirely by yolk and central nucleus (CNV), mature vitellogenic oocytes with cytoplasm filled entirely by yolk and showing germinal vesicle break down (GVBD), and atretic oocyte (AT).

### Blood sampling and steroids assays

Blood was collected at the moment of each hormonal doses and at the time of ovulation. Blood was collected by puncturing the caudal vein with heparinized syringes (Liquemine, Roche, Rio de Janeiro, RJ, Brazil) and needles. Blood was centrifuged at 1300 g for 10 minutes. The plasma was separated into aliquots and frozen at -80°C for the subsequent 17β-estradiol (E_2_) and 17α-hydroxyprogesterone (17α-OHP) assays. The plasma steroid level was measured through ELISA (Enzyme Linked Immunosorbent Assay) (E_2_ and 17α-OHP: Interteck, Virginia, USA). Plasma samples were run in duplicate with an acceptable limit of < 20.0 for the intra-assay coefficients of variation ( Brown *et al*., 2004 ). Absorbance measurements were collected using a microplate reader (Molecular Devices, CA, USA).

### Statistical analysis

Data normality was verified using the Cramer-von Mises test. Homoscedasticity was checked through the Fmax test. ANOVA test was used to analyze all parameters of reproductive performance, except for the percentage of spawning, which was analyzed through the Chi - square test (X^ 2^). The volume density was analyzed by comparing different treatments with a one-way analysis of variance (ANOVA). In order to analyze the gonadal steroids, two-way ANOVA for repeated measures was used. The Tukey’s test was used as a post hoc analysis. A threshold of P < 0.05 was set to infer statistical significance. All statistical analyses were based on Zar (1999) .

## Results

### Reproductive performance

The latency period was significantly higher for the CPE (18.50 hours) than for both mGnRHa treatments (3.5 µg/kg^−1^: 15.40 hours and 5 µg/kg^ −1^: 15.20 hours) (P = 0.03, Table 1 ). The percentage of spawning (P = 0.26, Table 1 ), absolute fecundity (P = 0.15, Table 1 ) and GSI (P = 0.58, Table 1 ) values were similar for all the CPE and mGnRHa treatments. Concerning the reproductive performance, the fertility rate for the CPE (23.6%) was markedly higher than those for the mGnRHa treatments (3.5 µg/kg^−1^: 1.4% and 5 µg/kg^−1^ : 0%) (P = 0.012, Table 1 ). On the other hand, there were no significant differences in hatching rate among treatments (P = 0.287, Table 1 ).

**Table 1:** Average values (± Standard error) of the reproductive performance of female *L. elongatus* undergoing hormonal induction.

Treatments	Latency period (h)	Spawning rate (%)	Absolute fecundity (oocyte/fish)	GSI (%)	Fertility rate (%)	Hatching rate (%)
CPE	18.50 ± 0.20^a^	80.00 ± 25.08^a^	84.334 ± 32.98^a^	16.49 ± 0.58^a^	23.60 ± 9.40^a^	1.3 ± 1.0^a^
mGnRHa (3.5 µg/kg^−1^)	15.40 ± 0.11^b^	100.00 ± 0.00^a^	97.239 ± 12.45^a^	12.80 ± 2.98^a^	1.40 ± 0.60^b^	0.4 ± 0.2^a^
mGnRHa (5.0 µg/kg^−1^)	15.20 ± 0.09^b^	100.00 ± 0.00^a^	95.338 ± 15.23^a^	11.58 ± 3.67^a^	0 ± 0 ^b^	0 ± 0 ^a^

CPE: carp pituitary extract and mGnRHa: mammalian gonadotropin-releasing hormone analogue. Different letters indicate differences between treatments (P < 0.05).

### Volume density of oocytes from ovaries collected at the time of ovulation

**Figure 1:**
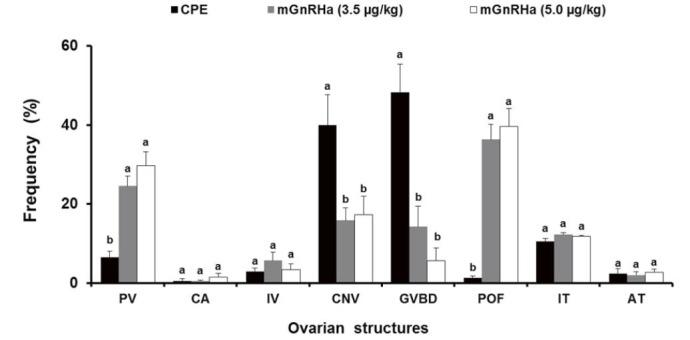
Percentage average values (± Standard error) of the volume density of different types of ovulated ovarian oocytes collected at the time of ovulation (n = 5 per treatment). Different letters indicate significant difference between treatments (P < 0.05). Oocytes: PV (previtellogenic); CA (cortical alveoli); IV (immature vitellogenic); CNV (vitellogenic oocytes with central nucleus); MNV (mature vitellogenic oocytes with migrated nucleus); GVBD (mature vitellogenic oocyte with germinal vesicle breakdown); POF (postovulatory follicle); AT (atretic) and TI (interstitial tissue). CPE: carp pituitary extract; mGnRHa: gonadotropin-releasing hormone.

**Figure 2:**
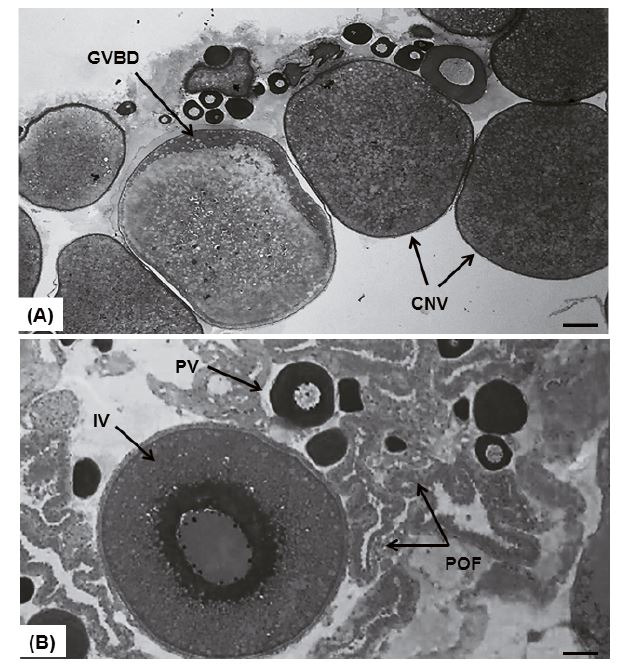
(A) Photomicrographs of cross sections of the ovary of *L. elongatus* females induced with CPE containing large amount of GVBD and CNV oocytes retained in post-spawning ovaries. (B) Photomicrographs of cross sections of the ovary of *L. elongatus* females induced with mGnRHa containing large amount of POF.

The volume density of the remaining PV in the ovaries after spawning was significantly higher for the mGnRHa treatments (3.5 µg/kg^−1^: 24% and 5 µg/kg ^−1^: 29%) compared to the CPE treatment (6.5%) (P < 0.0001, Fig. 1 and 2B ). The volume density of CA (P = 0.30, Fig. 1 ), IV (P = 0.42, Fig. 1 ), AT (P = 0.90, Fig. 1 ), and IT (P = 0.87, Fig. 1 ) were similar among treatments. The volume density of CNV was higher for the CPE treatment (39%) compared to the mGnRHa treatments (3.5 µg/kg^−1^: 15% and 5 µg/kg^−1^: 17%) (P = 0.0045, Fig. 1 and 2A ). Similarly, the volume density of GVBD was higher for the CPE treatment (48%) compared to the mGnRHa treatments (3.5 µg/kg^−1^: 14% and 5 µg/kg^ −1^: 5%) (P < 0.0001, Fig. 1 and 2A ). On the other hand, the volume density of POF was higher for the mGnRHa treatments (3.5 µg/kg ^−1^: 36% and 5 µg/kg^−1^: 39%) compared to the CPE treatment (1%) (P < 0.0001, Fig. 1 and 2B ).

### Gonadal steroids

Plasma E_2_ levels were similar among treatments before hormonal induction (P = 0.30, Table 2 ). However, during ovulation, in the mGnRHa treatments, levels were higher (3.5 µg/kg ^−1^: 132.06 pg mL^−1^ and 5 µg/kg^−1 ^: 150.60 pg mL^−1^) than in the CPE one (74.17 pg mL^−1 ^) (P = 0.02, Table 2 ). By assessing plasma levels of E_2_ during different sampling times within the same treatment, we could observe that in the CPE induced group there was a decrease between the first dose (129.27 pg mL^−1^) and ovulation (74.17 pg mL^−1 ^) (P = 0.03, Table 2 ). However, there was no significant difference in E_2_ levels between the time of the first dose (3.5 µg/kg^−1^: 154.45 pg mL^−1^ and 5 µg/kg^−1^: 127.82 pg mL^−1^) and ovulation (3.5 µg/kg^−1^: 132.06 pg mL^−1^ and 5 µg/kg ^−1^: 150.60 pg mL^−1^) in the mGnRHa treatments (P = 0.12, Table 2 ).

**Table 2:** Average values (± Standard error) of plasma levels of estradiol (E_2_ ) and 17α – hydroxyprogesterone (17α – OHP) at different times during hormonal induction.

Treatments		Period		
E_2_		First dose (pg mL^-1^)	Second dose (pg mL ^-1^)	Ovulation (pg mL ^-1^)
CPE		129,27 ± 26.26^aB^	132.15 ± 15.67^bB^	74.17 ± 28.96^aA^
mGnRHa (3.5 µg/kg^−1^)		154.45 ± 13.54^aA^	-	132.06 ± 15.87^bA^
mGnRHa (5.0 µg/kg^−1^)		127.82 ± 20.91^aA^	-	150.60 ± 10.75^bA^
17α – OHP				
CPE		198.08 ± 9.87^aB^	201.15 ± 5.22^bB^	229.87 ± 15.82^aA^
mGnRHa (3.5 µg/kg^−1^)		189.10 ± 29.05^aB^	-	376.04 ± 24.37^bA^
mGnRHa (5.0 µg/kg^−1^)		153.72 ± 64.89^aA^	-	401.10 ± 8.45^bA^

CPE: Carp pituitary extract and mGnRHa: mammalian Gonadotropin-releasing hormone analogue. Different letters indicate differences between treatments (P < 0.05). Different lowercase letters indicate differences between different treatments for the same periods and different capital letters indicate differences between the same treatment in different periods (P < 0.05).

Plasma levels of 17α - OHP in all CPE and mGnRHa (3.5 and 5 µg/kg^−1 ^) induced fish were similar during the first dose (P = 0.798, Table 2 ). However, during ovulation, in the mGnRHa treatments, levels were higher (3.5 µg/kg ^−1^: 376.04 ng mL^−1^ and 5 µg/kg^−1 ^: 401.10 ng mL^−1^) than in the CPE one (229.06 ng mL^−1 ^) (P = 0.03, Table 2 ). By assessing plasma levels of 17α - OHP during different sampling times within the same treatment, we could observe that, in all treatments, levels reached a peak at the time of ovulation (P = 0.0002, Table 2 ).

## Discussion

In the present study, we demonstrated that the treatments with low mGnRHa doses were more potent for inducing final maturation and ovulation in comparison with the conventional CPE protocol, as evidenced by the statistically higher values of GVBD and POF in post-spawning ovaries, as well as the higher gonadal steroid levels (at the time of ovulation); however, none of the treatments provided viable embryos.

Although the use of conventional doses of CPEs has already been shown to provide *L. elongatus* ovulation and viable embryos ( Sato *et al*., 2000 ), the low embryo viability obtained in all treatments in the present study (compared to Sato’s study) may be related to the use of different conditions and broodstock in each experiment, but mainly to the use of wild breeders herein. In this concern, it is known that the effects of broodfish on gamete quality remain poorly documented and there is a complete lack of information on the effect of the domestication of wild breeders on their reproductive performance ( Bobe and Labbé, 2010 ). It is also known that environmental control of gamete quality is far from being fully understood, especially the effect of external factors and broodstock management conditions on gamete quality ( Bobe and Labbé, 2010 ). Moreover, we must emphasize that, in the study published by Sato et al. (2000) , a large variation in the proportion of eggs retained by females and a relatively low rate of ovulation was reported, which can easily be replicated for different studies ( Hainfellner *et al*., 2012a ; Criscuolo–Urbinati *et al*., 2012 ; Pereira *et al*., 2017 ), using different broodstock, maintained under different conditions and performed in different years (with different climate characteristics).

The consistency in the results obtained using CPE and especially mGnRH in South American migratory fish is very far from being a reality. Induced ovulation with ovaprim (single dose: 10 µg Salmon Gonadotropin Releasing hormone analog (sGnRHa) kg^-1^ + 5 mg domperidone kg^-1^) has been very successful in *Colossoma macropomum*, but provided low quality embryos when compared to CPE ( Acuña and Rangel, 2009 ). In a congener species, *Leporinus macrocephalus*, low doses of mGnRHa (7 μg mGnRH kg-1 + 10 mg kg-1 metoclopramide) provoked ovulation, but not viable embryos ( Pereira *et al*., 2017 ). The induced ovulation failed in *Rhamdia quelen* (two doses: 2 µg mGnRHa kg^-1^ + 1 mg metoclopramide kg^-1^ and 20 µg mGnRHa kg^ -1^ + 10 mg metoclopramide kg^-1^ metoclopramide) ( Carneiro and Mikos, 2008 ). When applied to *Piaractus mesopotamicus*, *Brycon orbygnianus* and *Prochilodus lineatus,* busserelin acetate led to ovulation, but no viable embryos were obtained after fertilization ( Paulino *et al*., 2011 ). In addition, different from most South American rheophilic species, viable embryos and successful ovulation can be unusually obtained with very low doses of CPE (0.5mg kg^-1^ (with successive doses every six hours, if necessary)) in *Schizodon fasciatus* ( Lopes and Leal, 2010 ). Taken together, these findings indicate that *L. elongatus*, as well as other South American migratory species, do not respond properly (due to completely unknown reasons) to hormonal induction, especially with GnRH, which provides inconstant and very heterogeneous results of difficult reproducibility, especially if we consider that the doses applied in this study (3.5 or 5.0 μg mGnRH kg^-1^ + 10 mg kg^-1^ metoclopramide) and for the congener *L. macrocephalus* (7 μg mGnRH kg ^-1^ + 10 mg kg ^-1^ metoclopramide) which did not provide viable embryos either ( Pereira *et al*., 2017 ) were much lower than the GnRHa-implant doses reported as a cause for overstimulation in other fish species (50-100 kg^-1^) ( Mañanos *et al*., 2002 ; Rosenfeld *et al*., 2012 ).

According to those authors, the excessive secretion level of LH is one of the factors that may potentially affect the quality of eggs, promoting an overstimulation. In the present study, we did not evaluate LH levels, but considering the gonadal steroid levels evaluated, we emphasize that the CPE treatment was the only one to present a survival of the embryos (23.60 ± 9.40%) 5 hours after fertilization and, coincidently, that treatment was the only one to present a reduction of E_2_ at the time of ovulation, which remained similar over time for the mGNRHa treatments. In this concern, future approaches that correlate maternal plasma E_2_ levels with embryonic survival rates would be necessary to evaluate a possible maternal E_2_ transfer to eggs which may cause toxicity to them. It has already been demonstrated that unbalanced maternal plasma reproductive hormones are transferred to eggs ( Hwang *et al*., 1992 ; Mylonas *et al*., 1994 ) and are potential causes for embryo mortality in fish. There are also reports of higher E_ 2_ and 17α, 20β-Dihydroxy-4- pregnen-3-one (17α, 20β-DHP) levels in non-viable eggs in relation to viable eggs ( Feist *et al*., 1990 ). Furthermore, we observed excessive levels of gonadal steroids, concerning 17OHP, in all treatments at the time of ovulation, when compared to the levels at the first (or single) dose, which may have been the cause for an eventual toxicity and or overstimulation, since the parameters of water used in this experiment were adequate and neither explain the embryo mortality nor the the differential fertility rates found for the three treatments used. Although expected, since 17-OHP is the precursor of 17a, 20b-DHP named “maturation inducing substance” (MIS), which promotes germinal vesicle breakdown and the ovulation in teleosts ( Lubzens *et al*., 2010 ; Ogiwara *et al*., 2013 ; Hagiwara *et al*., 2014 ), plasma concentrations of 17-OHP for the mGNRHa treatments were higher than those for the CPE treatment, at the time of ovulation.

In conclusion, the use of mGnRHa associated with metoclopramide was more potent for inducing the *L. elongatus* ovulatory process when compared to CPE, as it significantly increased the FPO volume density and gonadal steroid levels at the time of ovulation. However, the results obtained here reinforced the existing hypothesis of an ovarian hyperstimulation with consequent egg toxicity due to the hormonal treatment in this rheophilic species and other South American ones, that must be addressed in futures studies. Recent reports on embryonic mortality obtained in spawning induced South American migratory species ( Acuña and Rangel, 2009 ; Carneiro and Mikos, 2008 ; Paulino *et al*., 2011 ; Pereira *et al*., 2017 ), coupled with the need for hormone-inducing such species in order to obtain embryos, point out to an immediate need for species-specific studies (probably *in vitro* ) in order to confirm which substances used in hormone therapies (and their derivatives), as well as which levels are indeed toxic to the eggs. This aspect needs to be addressed for the development of aquaculture in countries which depend on these species, since rheophilic fish are known to be among the most important fish for commercial purposes in South American countries ( MPA 2013 ; FAO 2014 ) and the constant and predictable supply of fingerlings is imperative for the consolidation of the use of any species for aquacultural purposes.
